# Patterns of domestication in the Ethiopian oil-seed crop noug (*Guizotia abyssinica*)

**DOI:** 10.1111/eva.12256

**Published:** 2015-04-13

**Authors:** Hannes Dempewolf, Misteru Tesfaye, Abel Teshome, Anne D Bjorkman, Rose L Andrew, Moira Scascitelli, Scott Black, Endashaw Bekele, Johannes M M Engels, Quentin C B Cronk, Loren H Rieseberg

**Affiliations:** 1 Department of Botany and Biodiversity Research Centre, University of British ColumbiaVancouver, BC, Canada; 2 Ethiopian Institute of Agricultural Research, Holetta Agricultural Research CentreAddis Ababa, Ethiopia; 3 Department of Plant Breeding, Swedish University of Agricultural SciencesAlnarp, Sweden; 4 Department of Geography and Biodiversity Research Centre, University of British ColumbiaVancouver, BC, Canada; 5 College of Natural Sciences, Addis Ababa UniversityAddis Ababa, Ethiopia; 6 Bioversity InternationalRome, Italy; 7 Department of Biology, Indiana UniversityBloomington, IN, USA

**Keywords:** agriculture, compositae, crop improvement, domestication, gene flow, genetic resource conservation, local adaptation

## Abstract

Noug (*Guizotia abyssinica*) is a semidomesticated oil-seed crop, which is primarily cultivated in Ethiopia. Unlike its closest crop relative, sunflower, noug has small seeds, small flowering heads, many branches, many flowering heads, and indeterminate flowering, and it shatters in the field. Here, we conducted common garden studies and microsatellite analyses of genetic variation to test whether high levels of crop–wild gene flow and/or unfavorable phenotypic correlations have hindered noug domestication. With the exception of one population, analyses of microsatellite variation failed to detect substantial recent admixture between noug and its wild progenitor. Likewise, only very weak correlations were found between seed mass and the number or size of flowering heads. Thus, noug's ‘atypical’ domestication syndrome does not seem to be a consequence of recent introgression or unfavorable phenotypic correlations. Nonetheless, our data do reveal evidence of local adaptation of noug cultivars to different precipitation regimes, as well as high levels of phenotypic plasticity, which may permit reasonable yields under diverse environmental conditions. Why noug has not been fully domesticated remains a mystery, but perhaps early farmers selected for resilience to episodic drought or untended environments rather than larger seeds. Domestication may also have been slowed by noug's outcrossing mating system.

## Introduction

Domestication is most accurately described as a process rather than an event (Zeder et al. 2006), and cultivated plants differ widely in their level of domestication. Some strongly domesticated crops, such as rice, wheat, corn, tomato, and sunflower, are substantially different from their wild ancestors. They often differ in traits that contribute to the ease of harvesting, such as reduced shattering, a more determinate growth habit (or increased apical dominance), larger inflorescences, larger seeds or fruits, loss of seed dormancy, reduction in nonpalatable substances in edible parts, changes in photoperiod sensitivity, and synchronized flowering time (Harlan et al. 1973; Koinange et al. 1996; Doebley et al. 2006; Purugganan and Fuller 2009). Other crops, however, including the Ethiopian cereal t'ef (*Eragrostis tef*), known as the world's smallest grain, and the Andean tuber crop yacon (*Smallanthus sonchifolius*), as well as many other locally important crops, show much weaker signs of domestication (Gepts 2004; D'Andrea 2008; Dempewolf et al. 2008). Possible explanations include variation in the type and strength of artificial selection pressures during domestication, interactions between natural and artificial selection, the length and duration of the domestication period, variation in the genetic architecture of the crop and its progenitor, variation in mating system, and varying degrees of gene flow with wild progenitors (Hillman and Davies 1990; Gepts 2004; Burger et al. 2008; Dempewolf et al. 2008). Here, we ask why noug (*Guizotia abyssinica* (L.f.) Cass.), a Compositae oilseed crop from Ethiopia (Hiremath and Murthy 1988), appears to be considerably less domesticated than sunflower (*Helianthus annuus* L.), a closely related seed crop also in the Compositae, which is thought to have been domesticated in the East-Central United States (Blackman et al. 2011).

It is unclear when noug was first cultivated, although there is some archaeobotanical evidence that suggests it was present in the Aksumite period (800 B.C.-A.D. 700) and was therefore likely first domesticated in pre-Aksumite times (Boardman 1999, 2000). Some authors even suggest noug was domesticated earlier than 3000 BC (Hiremath and Murthy 1988). At present, noug is grown primarily in Ethiopia, Eritrea, and India. The crop is popular with small-scale farmers in Ethiopia as it grows well in adverse conditions such as water-logged soils and produces reasonable yields under low-input conditions. It is well embedded into the traditional planting cycle and produces an edible oil that is highly sought after on the domestic market (Getinet and Sharma 1996).

Despite its importance to developing countries in East Africa, we know little about the domestication history of this species. Noug has been described as semidomesticated (Dempewolf et al. 2008); the crop is self-incompatible and highly branched, and flowering heads and seeds are less than one-tenth the size of sunflower, its closest oil-seed crop relative (Funk et al. 2009). Hence, unlike sunflower, noug does not exhibit strong signs of artificial selection and bears much greater resemblance to its wild relatives (Geleta 2007) than does sunflower to its wild relative. Despite their phylogenetic proximity within the Compositae, sunflower has responded to human selection pressure quite differently from noug; cultivated sunflowers, unlike their wild progenitors, are single-stemmed and often single-headed, do not shatter, and have much larger seeds (Burke et al. 2002). As both noug and sunflower are cultivated for their oil, one might expect similar artificial selection pressures in the two species, but this does not appear to be the case. Because sunflower and noug are so closely related—both are members of the Heliantheae tribe—comparisons between the two species can inform our understanding of general domestication patterns and processes.

In this study, we investigate two core hypotheses that might explain why noug remains semidomesticated despite thousands of years of cultivation: (i) crop–wild gene flow and (ii) unfavorable phenotypic correlations.

Noug is not known to occur in the wild and noug's closest relative *Guizotia scabra* ssp. *schimperii* (Sch.Bip. ex Walp.) J.Baagøe is thought to be its progenitor (Hiremath and Murthy 1988; Dagne 1994; Geleta 2007). As previously has been shown, and we confirm in this study, *Guizotia scabra* ssp. *schimperii* has smaller seeds than noug (Geleta 2007) but is also multibranched, shatters in the field, and exhibits differential maturity. It is a common and widespread species in many parts of Ethiopia, providing ample opportunity for gene flow between the two species (Geleta et al. 2010). Because *G. scabra* ssp. *schimperii* can produce fertile hybrids with noug (Dagne 1994), widespread introgression between the two species may have prevented full domestication of the latter. We test this hypothesis using a set of simple-sequence repeat (SSR) markers, also known as microsatellites, to estimate population structure and levels of admixture between noug and *G. scabra* ssp. *schimperii*.

In seed crops, yield may be enhanced by selection for increased seed size, increased seed number, or both. However, seed size often is negatively correlated with seed number, so selection on one trait may lead to reductions in the other. This may be due to genetic factors such as the pleiotropic effects of the same genetic locus or linkage disequilibrium among underlying loci, or to natural or conscious selection against unfit trait combinations (Falconer and Mackay 1996). In sunflower, for example, because of negative genetic correlations between seed size and branching (Tang et al. 2006), sunflowers with large seeds have fewer flowering heads (and fewer seeds) than plants with small seeds. Thus, a possible explanation for the atypical domestication syndrome in noug is that early farmers selected mainly for an increase in number of seeds or flowering heads, which inhibited the evolution of larger heads and larger seeds due to negative phenotypic correlations. Such negative phenotypic correlations are of considerable relevance to breeders; as such, plant-architectural constraints, if present, can be a major hindrance to targeted crop improvement efforts. We test this hypothesis by analyzing phenotypic correlations among key traits in common garden experiments at two sites in Ethiopia.

Lastly, we investigate the level and partitioning of genetic diversity between different noug accessions and determine whether there are any signs of local genetic structure across noug's range in Ethiopia. When combined with the phenotypic results, this information allows us to examine the importance of local genetic differentiation, perhaps due to adaptation to local environmental conditions during noug's history of domestication—an aspect that is also of relevance to future germplasm collection missions, which aim to capture a maximum amount of phenotypic diversity.

## Materials and methods

**Figure 1:**
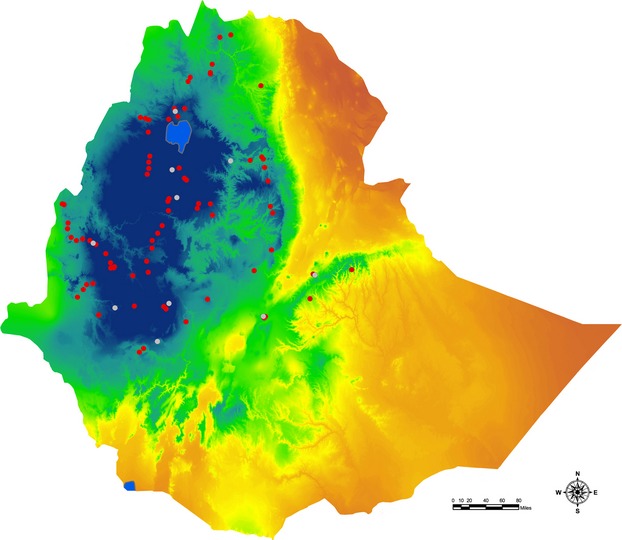
A map of Ethiopia showing the location for each collection of noug (in red) and of *G. scabra* ssp. *schimperii* (in gray). The map also shows precipitation gradients across the country. Dark blue areas indicate areas with high precipitation during the wettest quarter, green areas indicate intermediate rainfall during the wettest quarter, and areas shown in yellow and red indicate low rainfall during this period.

Collections of noug and of noug's putative progenitor, *Guizotia scabra* ssp. *schimperii*, were made across an altitudinal gradient of approximately 2300 meters, ranging from semi-arid climate conditions at 896 meters above sea level to subalpine conditions at 3199 meters above sea level. From each field of noug (or population of *Guizotia scabra* ssp. *schimperii)*, approximately 50 individuals were sampled at random and the seeds from each individual plant were kept separate. Samples from each field of noug or stand of *Guizotia scabra* ssp. *schimperii* were considered a population for the purpose of this manuscript. Five administrative regions in Ethiopia (i.e. Amhara, Oromia, Tigray, Southern Nation Nationalities and Peoples, and Beni-Shangul Gumuz) were covered during the harvest season. A GPS device was used to record the precise geographical location of each collection (Fig.1).

The noug diversity collected in the field was characterized in two common garden experiments to evaluate phenotypic trait variation. These common gardens were located at the Holetta (2390 m altitude, 35 km west of Addis Ababa) and Ginchi (2200 m altitude, 85 km west of Addis Ababa) experimental stations operated by the Ethiopian Institute of Agricultural Research (EIAR). Although the two sites are only 38 km apart, Ginchi is at a lower altitude and has soils with higher clay content than Holetta. The soil types and elevation of both sites are typical of those found at the collection localities. Precipitation during the growing season (the wettest quarter) at the Ginchi and Holetta sites in 2009 was 587 mm and 525 mm, respectively. This is within the range of average wettest quarter precipitation for the collection localities (344–1135 mm). Seeds collected from the ∼50 individuals from each field were pooled before sowing. Four blocks containing each of the 29 accessions in randomized locations were sown with 0.3 m distance between two rows of the same accessions and 0.6 m distance between plots of different accessions. Stand count at establishment was assessed per plot at approximately 4 weeks after planting and varied between one and 222 individuals at the Ginchi site (51 individuals on average), and between 5 and 490 individuals at the Holetta site (222 individuals on average), depending on survival success during the establishment phase. The Ginchi common garden was damaged by a frost, which increased the variance in survivorship within the Ginchi site, as well as between the two sites. If fewer than ten individuals from a population survived to maturity, the accession was excluded from the analysis. The accessions at Holetta were planted on the 15th of July 2009 and in Ginchi on the 17th of July 2009. The following traits were phenotyped on ten mature plants, which were selected at random from each accession: leaf width, leaf length, stem diameter, number of heads, number of primary branches, number of secondary branches, number of stem leaves, plant height, and head size. Individual plants were bagged before seeds matured to minimize the loss of seeds through shattering. The number of seeds per plant, number of seeds per head, and the mass of 1000 seeds (used as a proxy for seed size) were measured after harvest. In addition, flowering time was assessed for each accession as the number of days until 50% of plants per plot flowered.

We obtained environmental data for all collecting locations using the publicly available Worldclim/Bioclim dataset (http://www.worldclim.org/bioclim), which contains a set of 19 environmental variables, including precipitation and temperature data. In addition, we compiled altitudinal and latitudinal data for each collection point. Statistical analyses of the phenotypic data were carried out in *R v. 2.10.0* (R Development Core Team 2011). Where necessary, variables were transformed using square or square root transformations to meet the assumptions of normality and homogeneity of variance. Correlations between traits were determined using Pearson's partial correlations. Partial correlations were used to control for the effect of differing stand densities at maturity, which varied substantially between the two common garden locations due to a severe frost and subsequent high mortality at one site. For correlations between individual and pooled traits (1000 seed mass and day of 50% flowering), we used the population mean of the individual trait. Correlations were calculated for each site separately, and *P*-values were Bonferroni-corrected to compensate for multiple comparisons. We conducted an ordination of the phenotypic trait data from both common garden sites using a nonmetric multidimensional scaling (NMDS) analysis with Bray–Curtis dissimilarity in the R package ‘vegan’ (v. 1.7-8) (Oksanen et al. 2010). Trait values were averaged for each population within each site. We then estimated the correlation between the multivariate trait data and the environmental variables (Table1) using the *envfit* function (also in the ‘vegan’ package), which fits environmental vectors onto the ordination and estimates the strength (*R*^2^) and significance of the relationship with *P*-values based on 1000 random permutations of the data.

**Table 1:** Fit of environmental variables onto the ordination of traits

	NMDS 1	NMDS 2	*R* ^2^	*P*-value
Latitude	−0.924	−0.3825	0.0006	0.94
Longitude	−0.0718	0.9974	0.0146	0.3213
Elevation	0.8577	−0.5142	0.0007	0.946
Mean temperature of coldest quarter	−0.3771	0.9262	0.0025	0.8186
Mean temperature of warmest quarter	−0.1985	0.9801	0.005	0.6567
Annual precipitation	0.061	−0.9981	**0.0505**	**0.012**
Precipitation of wettest month	0.0257	−0.9997	**0.0740**	**0.001**
Precipitation of driest month	0.0433	0.9991	0.0093	0.4653
Precipitation seasonality	−0.4665	−0.8845	0.0011	0.9155
Precipitation of wettest quarter	0.0352	−0.9994	**0.0945**	**0.0005**
Precipitation of driest quarter	0.0328	0.9995	0.0072	0.5512
Precipitation of warmest quarter	0.3295	0.9442	0.0024	0.8171
Precipitation of coldest quarter	0.0327	−0.9995	**0.0397**	**0.045**
Annual mean temperature	−0.2344	0.9722	0.0043	0.6972
Mean diurnal range	−0.1861	0.9825	0.0117	0.3958
Isothermality	−0.1628	0.9867	0.006	0.6292
Temperature seasonality	0.0329	0.9995	0.0215	0.1674
Max temperature of warmest month	−0.2059	0.9786	0.005	0.6592
Min temperature of coldest month	−0.2316	0.9728	0.0022	0.8491
Temperature annual range	−0.169	0.9856	0.0033	0.7766
Mean temperature of wettest quarter	−0.1947	0.9809	0.0095	0.4693
Mean temperature of driest quarter	−0.3341	0.9425	0.0013	0.9075

The values in the first two columns give a vector describing direction of the environmental gradient, relative to the two NMDS axes. *R*^2^ values and *P*-values indicate the strength and significance of the relationship. Bold values are significant at *P* < 0.05.

To test whether significant effects could be the product of spatial differentiation, we performed partial Mantel tests on Euclidean geographic, precipitation, and NMDS distances. Using the *mantel* function of the ‘ecodist’ package, the effect of precipitation distance on trait distance while controlling for spatial distance was estimated and tested with 1000 permutations and one-sided tests.

We used linear mixed effects models or generalized mixed effects models with a Poisson error distribution (for those variables that were counts) to determine whether each trait changed significantly with precipitation at the population origin. We included the precipitation during the wettest quarter (evaluated at each collection locality), common garden site, and stand density at maturity as fixed effects in the model, as well as interactions between all pairs of variables. Population was included as a random effect to account for the nestedness of individuals within each population, with random slopes for common garden site to allow populations to vary by site. Models were run using the lmer or glmer functions in the R package lme4. Nested models were compared using chi-squared likelihood ratio tests. Terms that did not improve the model fit were dropped from the model (*P* ≤ 0.05), and only the significant terms are reported. We used linear models for the two traits (day of 50% flowering and 1000 seed mass) that were measured at the population level.

A subset of 29 noug accessions as well as four populations of *G. scabra* ssp. *schimperi* were genotyped with a set of 16 microsatellite markers that had previously been developed for noug: GA003, GA012, GA029, GA035, GA081, GA082, GA107, GA108, GA117, GA138, GA139, GA150, GA156, GA162, GA182, and GA210 (Dempewolf et al. 2010). Genotyping was performed following the protocol described in Dempewolf et al. (2010), using between 14 and 23 individuals per population (average 19.3 individuals per population).

Microsatellite summary statistics were calculated, and analysis of molecular variance (amova) was performed in GenAlEx 6.5 (Peakall and Smouse 2006). Shannon's diversity indices, which partition diversity within and between populations, were used to compare the genetic diversity unique to each taxon (Sherwin et al. 2006), with calculations based on the natural logarithm. The information content of the data for ancestry estimation (*I*_*a*_), based on the noug–wild relative distinction, was estimated using *infocalc* version 1.1 (Rosenberg et al. 2003). We then employed a model-based clustering method using the software program *STRUCTURE* v. 2.3.2.1 (Pritchard et al. 2000; Falush et al. 2003; Hubisz et al. 2009) to assign individuals to a predefined number of groups, as well as to infer levels of admixture. The correlated allele frequency model with admixture permitted was used here. The burn-in period was set to 500,000 generations, and the number of MCMC generations was set to 4 000 000. Twenty independent runs were performed, and a range of one to 20 clusters (Ks) was explored in each run. We determined the optimal number of clusters by comparing the log-probability of the data among values of K and using the ‘Evanno’ method (Evanno et al. 2005), as implemented in *Structure Harvester* v. 0.6.5 (Earl and vonHoldt 2011), which estimates the optimal number of clusters (K) by inferring Delta K, which is the second-order increase in likelihood for each K (Evanno et al. 2005).

**Figure 2:**
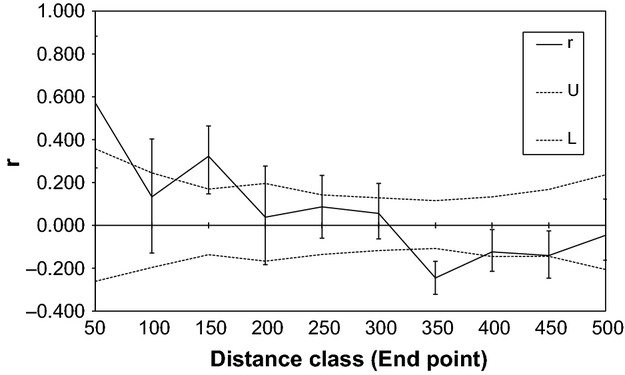
Summary of subpopulation-level spatial autocorrelation analysis with 50 km distance classes. The autocorrelation coefficient (*r*, solid line) for the distance class (in meters) was bias-corrected and tested against a null hypothesis of no spatial structure using both bootstraps (95% error bars) and permutations (95% confidence limits, gray dashed lines) with 1000 replicates.

To test for spatially structured genetic variation among noug accessions, spatial autocorrelation analysis was performed on genetic distances between subpopulations in GenAlEx 6.4 (Peakall and Smouse 2006; Smouse and Peakall, 1999). Multiple distance classes were tested and gave qualitatively similar results; only 50 km nonoverlapping distance classes are shown here (Fig.2). Permutation and bootstrap tests were conducted with 1 000 replicates in each case.

## Results

Contrary to expectations, Pearson's partial correlation analyses (Table2) failed to detect significant negative correlations between 1000 seed mass (used as a proxy for seed size) and head diameter, the number of flowering heads, and the number of seeds per plant. In fact, a significant positive correlation was observed between 1000 seed mass and head size at one of the common garden sites (Site 2, Ginchi). We also observed the expected significant positive correlations between the number of primary/secondary branches and the numbers of heads and seeds per plant at both sites.

**Table 2:** Phenotypic trait correlations of a selected number of traits related to plant architecture

	Height	Number of seeds per head	Head size	Number of heads	Number of sec. branches	Number of prim. branches	Seeds per plant	1000 Seed weight	Days to 50% flowering
SITE 1									
Height	–	0.024	0.0174	***0.1705***	***0.1251***	***0.143***	0.0505	0.2947	***0.4802***
Number of seeds per head	*P* = 0.498	–	0.0758	0.0083	0.049	0.0359	***0.3652***	0.0714	−0.0705
Head size	*P* = 0.5802	*P* = 0.0306	–	−0.0008	0.0409	0.0235	−0.0158	0.0666	−0.0954
Number of heads	*P < 0.0001*	*P* = 0.8127	*P* = 0.979	–	***0.355***	***0.6515***	***0.4413***	−0.0439	***0.6369***
Number of sec. branches	*P < 0.0001*	*P* = 0.1552	*P* = 0.1952	*P < 0.0001*	–	***0.332***	***0.2118***	0.145	***0.425***
Number of prim. branches	*P < 0.0001*	*P* = 0.3058	*P* = 0.4569	*P < 0.0001*	*P < 0.0001*	–	***0.368***	0.1743	***0.56***
Seeds per plant	*P* = 0.1404	*P < 0.0001*	*P* = 0.645	*P < 0.0001*	*P < 0.0001*	*P < 0.0001*	–	−0.0884	***0.3364***
1000 Seed weight	*P* = 0.08	*P* = 0.5293	*P* = 0.5772	*P* = 0.6985	*P* = 0.1995	*P* = 0.122	*P* = 0.4356	–	0.0031
Days to 50% flowering	*P < 0.0001*	*P* = 0.5343	*P* = 0.4	*P < 0.0001*	*P < 0.0001*	*P < 0.0001*	*P = 0.0023*	*P* = 0.9786	–
SITE 2									
Height	–	−***0.1831***	***0.1325***	***0.484***	***0.3923***	***0.357***	0.0485	0.1731	***0.647***
Number of seeds per head	*P < 0.0001*	–	−0.0235	−***0.1111***	−0.0743	−0.0481	***0.1373***	−0.0785	−0.2104
Head size	*P < 0.0001*	*P* = 0.5345	–	0.005	0.0861	0.05218	0.0143	***0.4582***	***0.4623***
Number of heads	*P < 0.0001*	*P = 0.0029*	*P* = 0.8803	–	***0.6466***	***0.4085***	***0.2406***	−0.1677	***0.4607***
Number of sec. branches	*P < 0.0001*	*P* = 0.0461	*P* = 0.0098	*P < 0.0001*	–	***0.5758***	***0.2098***	−0.2769	***0.5135***
Number of prim. branches	*P < 0.0001*	*P* = 0.198	*P* = 0.118	*P < 0.0001*	*P < 0.0001*	–	***0.2656***	−0.0463	***0.6261***
Seeds per plant	*P* = 0.1989	*P = 0.0009*	*P* = 0.7061	*P < 0.0001*	*P < 0.0001*	*P < 0.0001*	–	0.06441	0.0022
1000 Seed weight	*P* = 0.1247	*P* = 0.489	*P < 0.0001*	*P* = 0.1371	*P = 0.013*	*P* = 0.6832	*P* = 0.5728	–	0.2553
Days to 50% flowering	*P < 0.0001*	*P* = 0.0610	*P < 0.0001*	*P < 0.0001*	*P < 0.0001*	*P < 0.0001*	*P* = 0.9846	*P* = 0.0223	–

Pearson's partial correlation between traits after removing the effect of varying stand densities at both common garden sites. Numbers of above the diagonal are the correlation values, numbers below the black line are *P*-values. Bold and italicized numbers are significant after a Bonferonni correction for multiple comparisons was applied.

**Figure 3:**
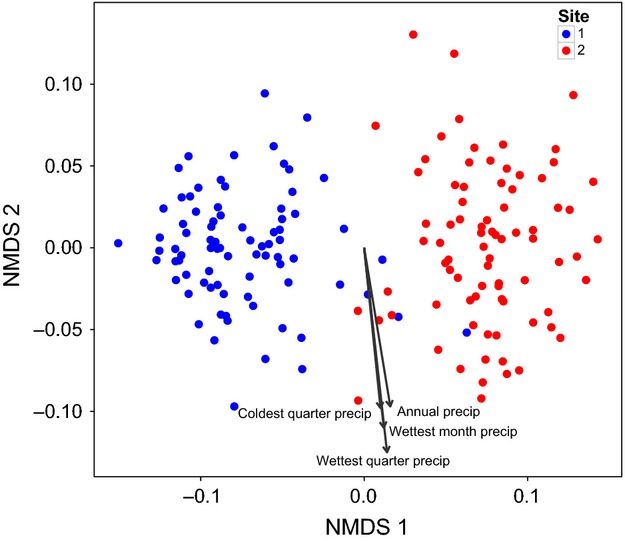
NMDS analysis of phenotypic data of both sites (red = Holetta Site 1 and blue = Ginchi Site 2) with environmental variables fitted onto the ordination. Each point represents the population-mean trait composition of one population at one site. The length of each arrow is proportional to the correlation between the variable and the ordination. The four precipitation variables: (i) precipitation of wettest quarter (*r*^2^ = 0.095, *P* < 0.001, (ii) precipitation of wettest month (*r*^2^ = 0.074, *P* < 0.001), (iii) annual precipitation (*r*^2^ = 0.051, *P* = 0.012), and (iv) precipitation of coldest quarter (*r*^2^ = 0.040, *P* = 0.045), are highly correlated with each other, which is why their vectors point in a similar direction.

**Figure 4:**
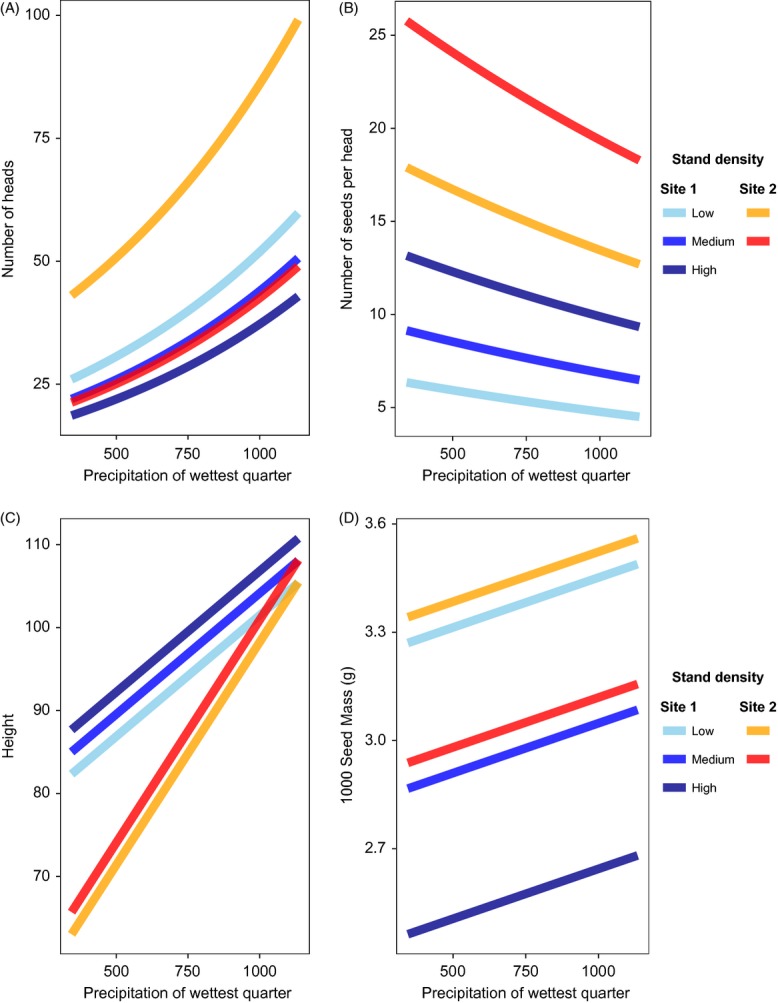
Predicted values from mixed model analyses relating precipitation of the wettest quarter to: (A) number of heads, (B) number of seeds per head, (C) height, and (D) 1000 seed mass (g). Predicted values are extracted from full model (non-significant terms also included); see Table3 for significance of each variable. Blue lines represent predicted values for Site 1, red lines represent predicted values for Site 2. Color shading corresponds to stand density, with darker shades representing higher stand density. No predicted values are shown for Site 2 at ‘high’ stand density because no population at Site 2 actually reached this density level.

The NMDS analysis shows that noug accessions cluster according to common garden site along the axis of most variation (NMDS1) (Fig.3). The spread of noug accessions along the second axis (NMDS2) correlates most strongly with the precipitation variables ‘precipitation of wettest quarter’ (*r*^2^ = 0.0945, *P* < 0.001), ‘precipitation of wettest month’ (*r*^2^ = 0.074, *P* < 0.001), ‘annual precipitation’ (*r*^2^ = 0.051, *P* = 0.012), and ‘precipitation of coldest quarter’ (*r*^2^ = 0.0397, *P* = 0.045, Table1). No other environmental variables were significantly correlated with the multivariate trait data. Although geographic distances between populations were correlated with both distances along NMDS2 (Mantel's *r *=* *0.113, *P *=* *0.027) and the differences between populations in the precipitation of the wettest quarter (Mantel's *r *=* *0.135, *P *=* *0.006), the correlation between trait and precipitation distances remained significant when controlling for geographic distance (partial Mantel's *r *=* *0.154, *P *=* *0.003). The results of the mixed effects model testing for differences among populations according to their native precipitation regime were significant for several traits, including leaf width, leaf length, stem diameter, number of heads, number of primary branches, number of secondary branches, height, head size, number of seeds per head, and days to 50% flowering (Table3, Fig.4). Three variables (leaf width, height, and head size) showed significant interactions between precipitation and common garden site. Only the number of seeds per plant and 1000 seed weight did not change significantly with precipitation regime.

**Table 3:** Phenotypic differences among noug accessions from different precipitation regimes

	Preciptation		Site		Stand density		Precipitation × site		Stand density × site	
	Direction	*P*-value	Direction (Site 2)	*P*-value	Direction	*P*-value	Direction	*P*-value	Direction	*P*-value
Leaf_width	+	NA	**−**	NA	**−**	***P***** = 0.0273**	**+**	***P***** = 0.0036**	n.s.	*P* = 0.9876
Leaf_length	**+**	***P***** = 0.0003**	+	NA	+	NA	n.s.	*P* = 0.1636	**+**	***P***** = 0.0222**
Stem_diam	**+**	***P***** = 0.0007**	**+**	***P***** = 0.0203**	n.s.	*P* = 0.3140	n.s.	*P* = 0.8897	n.s.	*P* = 0.2442
No_heads	**+**	***P***** < 0.0001**	+	NA	**−**	NA	n.s.	*P* = 0.1230	**−**	***P***** < 0.0001**
No_prim_br	**+**	***P***** < 0.0001**	**+**	***P***** = 0.0143**	**−**	***P***** < 0.0001**	n.s.	*P* = 0.1741	n.s.	*P* = 0.1762
No_sec_br	**+**	***P***** < 0.0001**	**+**	***P***** < 0.0001**	**−**	***P***** = 0.0001**	n.s.	*P* = 0.4633	n.s.	*P* = 0.4557
Height	+	NA	**−**	NA	**+**	***P***** = 0.0012**	**+**	***P***** = 0.0014**	n.s.	*P* = 0.7199
Head_size	**−**	NA	**−**	NA	+	NA	**+**	***P***** = 0.0007**	**−**	***P***** = 0.0035**
No_seeds_plant	n.s.	*P* = 0.2903	**+**	***P***** < 0.0001**	**+**	***P***** = 0.0312**	n.s.	*P* = 0.0986	n.s.	*P* = 0.2914
No_seeds_head	**−**	***P***** = 0.0104**	**+**	***P***** < 0.0001**	**+**	***P***** < 0.0001**	n.s.	*P* = 0.1185	n.s.	*P* = 0.1873
Day_50pc_flwr	**+**	***P***** < 0.0001**	**−**	***P***** = 0.0307**	n.s.	*P* = 0.1025	n.s.	*P* = 0.3816	n.s.	*P* = 0.7029
SeedWt1000	n.s.	*P* = 0.3271	n.s.	*P* = 0.6527	**−**	***P***** < 0.0001**	n.s.	*P* = 0.8817	n.s.	*P* = 0.4342

Fixed effects terms from the linear and generalized linear mixed models. Significance of the terms was determined by comparing nested models using chi-squared likelihood ratio tests. Bold values are significant at *P* ≤ 0.05. Non-significant terms were removed from the final model. The direction of the modeled relationship between each term and the trait of interest is given by (+) and (−) signs. Parameters that were not significant in the model are denoted by n.s.

The microsatellite loci displayed moderately high variation (Table4), but possible evidence of ascertainment bias: of 137 alleles, 53 were private in noug, compared with 7 in its wild relative (77 were shared). However, sampling was also more intensive at the population level in noug.

**Table 4:** Genetic marker summary statistics

	*G. abyssinica*		*G. scabra* ssp. *schimperii*	
	Mean	SE	Mean	SE
N	18.056	0.182	18.750	0.527
Na	4.108	0.081	3.479	0.204
Ne	2.164	0.041	2.132	0.124
I	0.881	0.019	0.831	0.058
Ho	0.398	0.010	0.341	0.033
He	0.463	0.009	0.457	0.031
UHe	0.478	0.010	0.470	0.032
*F*_is_	0.155	0.036	0.259	0.065
*F*_it_	0.191	0.035	0.290	0.066
*F*_st_	0.044	0.003	0.059	0.024

The statistics shown include N, the number of individuals genotyped; Na, the number of alleles; Ne, the effective number of alleles; I, Shannon's information index; Ho, the observed heterozygosity; He, the expected heterozygosity; UHe, an unbiased estimate of expected heterozygosity (Peakall and Smouse, 2012). *F*_is_, *F*_it_, and *F*_st_ are multi-allelic analogues of Wright's *F*-statistics. All statistics are averaged over populations and loci.

**Figure 5:**
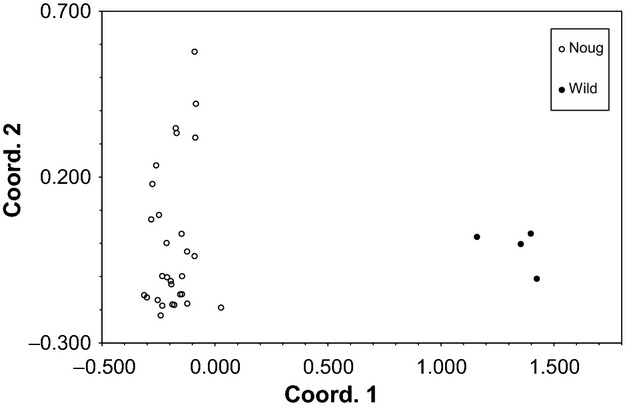
Principal coordinates analysis of noug and its putative progenitor based on microsatellite data.

Significant population structure occurred within both noug (*Φ*_PT_ = 0.058, *P *=* *0.001) and *G. scabra* ssp. *schimperii* (*Φ*_PT_ = 0.046, *P *=* *0.001). Overall, amova indicated that 6% of variation occurred among populations (*Φ*_PR_ = 0.061, *P *=* *0.001; *Φ*_PT_ = 0.397, *P *=* *0.001) and 36% between taxa (*Φ*_RT_ = 0.368, *P *=* *0.001). Shannon's diversity indices indicated approximately equal diversity within noug and its putative progenitor (mean ^*S*^*H*_*A*_ across loci of 0.989 and 0.987, respectively), despite significant differentiation at all loci (*P *<* *0.001, Table S2). Principal coordinates analysis also indicated differentiation between the taxa (Fig.5) along the first axis, which explained 72.0% of the variation (axis 2 explained 11.8%).

**Figure 6:**
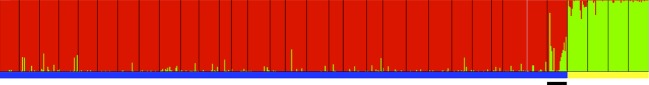
Assignment of individuals to two clusters (*k* = 2) by the program STRUCTURE. Accessions of noug are underlined in blue, whereas populations of noug's putative progenitor are underlined in yellow. The accession marked by a black bar is likely of hybrid origin.

*STRUCTURE* analyses revealed a genetic discontinuity between noug and its putative progenitor. For the dataset that included all 29 noug accessions and four populations of the wild relative, *G. scabra* ssp. *schimperii*, the log-probability of the data given the model increased sharply from *K* = 1 to *K* = 2, with small increases thereafter (Fig. S1a) and the ‘Evanno’ method (Evanno et al. 2005) estimated the optimal number of clusters (*K*) to be two (Fig. S1b). The *STRUCTURE* graph at *K* = 2 clearly assigns all populations of *G. scabra* ssp. *schimperii* (Fig.6) to one cluster, and all but one of the noug accessions to a second cluster. One noug accession (70011) appeared to contain multiple admixed individuals; however, only two individuals showed strong evidence of admixture based on the 90% probability intervals of their ancestry coefficients (Fig. S3), indicating that overall levels of recent introgression between noug and its wild relative are low.

Within noug, no clustering was detected using this method (Fig. S2). However, spatial autocorrelation analysis detected significant spatial structure among noug populations (Fig.2). Significant positive spatial autocorrelation occurred within 50 km and between 100 and 150 km. Congruent results were obtained for each of the distance class sizes that we applied, ranging from 30 to 80 km (not shown).

## Discussion

We tested the hypothesis that unfavorable phenotypic correlations have hindered noug domestication. Specifically, we asked whether plants with more flower heads and more seeds tend to have smaller flower heads and smaller seeds. Such negative phenotypic correlations are frequently evident in seed crops, including annual sunflowers, in which this phenotypic correlation is at least partially genetically based (Tang et al. 2006). In noug, however, a significant positive correlation was observed between seed mass and the number of seeds per plant. Correlations between seed mass and the number of seeds per head and the number of seeds per plant were not significant and showed different trends at the two different common garden sites. Thus, we found no evidence that unfavorable phenotypic correlations have constrained the evolution of seed size in noug. It can therefore be concluded that plant-architectural constraints to breeding for larger and more seeds in noug are minimal. A possible caveat is that we have analyzed phenotypic rather than genetic correlations, and it is possible that some of the variation underlying these phenotypes has a common genetic basis that could limit responses to selection despite the lack of negative phenotypic correlations.

We also tested whether introgression between noug and its putative wild progenitor is the cause for the observed similarity in phenotype between the two species. If this hypothesis were true, we would expect to see evidence of repeated introgression from the wild species into the domesticated species in our microsatellite analysis. In this analysis, however, evidence of recent introgression between noug and its closest wild relative and putative progenitor, *Guizotia scabra* ssp. *schimperii* (Figs5 and 6, Fig. S3), was limited to two noug individuals. Although these results do not preclude more widespread historical introgression, current levels of gene flow are too low to prevent genetic divergence, especially when driven by artificial selection.

Genetic divergence among noug accessions was surprisingly low relative to other crops. Many noug farmers attempt to save their own seed for sowing in the next year whenever possible, but they often run out of stored seed and will buy market seed instead—especially in years when noug production is low and saved seed is needed for personal consumption (Dempewolf, personal observation). Farmers also frequently exchange noug seeds with neighboring farmers, resulting in additional seed (and thus gene) flow between farms, even across large geographic distances. When asked about the level of diversity, farmers usually did not distinguish between different noug types, nor were they usually aware of noug diversity elsewhere (Dempewolf, personal observation). The apparent lack of awareness of diversity within noug stands in stark contrast to diversity recognized by farmers in many other crops (Jarvis et al. 2008).

Earlier studies have reported regional genetic differentiation within noug based on RAPD and AFLP markers (Geleta et al. 2006, 2008), which is consistent with the significant spatial genetic autocorrelation observed in our data. Nevertheless, only a small proportion of genetic variance (*F*_st_ = 0.044) occurred among noug populations. Genetic exchange between different noug accessions is facilitated by the fact that the crop has a self-incompatibility system and is obligately outcrossing (Geleta and Bryngelsson 2010). Crops with self-incompatibility systems are known to be more challenging to improve through breeding, as it is difficult to keep lines of interest ‘true to type.’ If an interesting phenotype is discovered in a certain accession, there is a high risk that the phenotype of interest will no longer be present in the next generation, as maintenance of the genetic identity of the line through selfing is not possible.

This may also present a barrier to farmer-led improvement efforts and may be the reason why none of the farmers we encountered claimed to actively improve or select for certain agronomic characteristics in noug. A lack of farmer-led improvement would also explain why most farmers were not aware of much noug diversity in terms of landraces or varieties. A lack of intensive improvement efforts by farmers during earlier phases of noug domestication might have also been a major cause for the crop's apparently ‘weak’ domestication syndrome.

The wild progenitor of sunflower is also self-incompatible, so why does the case of sunflower domestication differ in this respect from noug? One possibility is that self-compatibility evolves more easily in sunflower, as alleles that confer self-compatibility are not uncommon in wild sunflower populations, with frequencies ranging from 1 to 16% (Burke et al. 2002; E. Drummond and L.H. Rieseberg unpublished). However, no comparisons have been made with noug to determine the ease with which self-compatibility evolves in the two crops.

Our results, as well as conversations with noug farmers, indicate that an important focus for future breeding efforts should be the development and use of self-compatible lines. There also is a clear need to increase yield and to reduce the amount of seed lost because of asynchronous flowering and shattering. Our phenotypic analyses indicate that strong selection for increased seed size may be the most straightforward means of achieving the desired increases in yield.

Even though there is little genetic divergence among different noug accessions, there does appear to be some adaptation to different environmental conditions across noug's range. In the NMDS analysis (Fig.3), accessions clearly cluster according to the common garden site at which they were grown. Major variation is evident between sites, indicating that phenotypic plasticity plays an important role in explaining phenotypic differences between the two common garden sites and, by extension, much of the phenotypic diversity observed in farmer's fields across Ethiopia. However, none of the environmental variables considered here are correlated with the spread of data along the *x*-axis, implying that plasticity is not structured according to the environmental variation among source locations. The spread of the data along the *y*-axis, which explains the second largest proportion of variation in this NMDS analysis, is most strongly correlated with four precipitation variables (Fig.3). The results of the mixed model analyses (Table3) show that leaf width, leaf length, number of heads, number of primary branches, number of secondary branches, plant height, and days to 50% flowering are all traits that significantly differ between accessions that originated from different precipitation zones, even when grown in common gardens. This suggests that the level of precipitation during the growing season of noug may be a key environmental factor driving noug phenotypic diversity – an important aspect to consider in future collection missions that aim to capture the maximum diversity of the crop for conservation purposes. The results of the NMDS and mixed effects model analyses therefore provide some evidence for local adaptation to different precipitation regimes.

It will be important for noug breeders to be aware of the high levels of phenotypic plasticity of the crop, which requires that cultivars be tested in a wide variety of different environments. However, high levels of phenotypic plasticity also mean that even under strong seasonal environmental fluctuations, such as drought or flooding events, noug will likely still be able to produce some yield. Episodic drought events and variable climatic conditions have been characteristic in the region for several thousand years (reviewed in Harrower et al. 2010). Therefore, an alternative hypothesis which could help explain noug's phenotype is that farmers did not prioritize selection for seed size or other domestication traits as seen in sunflower, but rather (consciously or unconsciously) selected for a resilient crop that performs well under diverse conditions (Zohary 2004). This hypothesis has been suggested in the context of the domestication history of the Ethiopian cereal t'ef (*Eragrostis tef*), which is commonly recognized as the world's smallest grain and has a high level of branching and uncompacted panicles (D'Andrea 2008). A third alternative hypothesis is that the high levels of phenotypic plasticity are a consequence of the poorly developed domestication syndrome in noug rather than the cause of it. High levels of plasticity could be slowing down the response to artificial selection (Ancel 2000). Even though the experiments described here do not allow us to clearly distinguish between these three hypotheses, the results of our common garden experiments do suggest that phenotypic plasticity plays a major role in explaining trait variation in noug. To determine whether increases in phenotypic plasticity have accompanied noug domestication, we must first compare noug productivity with that of its putative progenitor in a series of common garden experiments across diverse environments.

Neither introgression between noug and its progenitor nor unfavorable phenotypic correlations were found to be substantial barriers to noug domestication. The crop's self-incompatibility system and extensive gene flow among farmer's fields likely contributes to noug's status as a semidomesticated crop and to its apparent lack of selection and diversification attempts by farmers. Phenotypic plasticity appears to be an important factor in explaining trait variation observed in noug in farmer's fields, and plasticity may permit the crop to produce reasonable yields even in diverse conditions. There is also evidence of adaptation to different precipitation regimes. We propose a series of common garden experiments to investigate the alternative hypothesis that domestication in noug has focused more on resilience to diverse environmental conditions rather than a sunflower-like domestication syndrome.

## Data archiving statement

Genetic and Phenotype data available from the Dryad Digital Repository: http://dx.doi.org/10.5061/dryad.gs352.

## Supporting Information

**Figure S1.** Comparison of STRUCTURE analysis of microsatellite data for the dataset that included all 29 noug accessions and 4 populations of the wild relative, *G. scabra* ssp *schimperii*. **Figure S2.** Comparison of model fit for different numbers of clusters in STRUCTURE analysis for 29 noug populations. **Figure S3.** Probability intervals for STRUCTURE analysis. **Table S1.** Summary statistics for noug and its putative wild progenitor, *G. scabra* ssp *schimperii*. **Table S2.** Summary of locus-specific analyses of diversity and divergence.
