# Supplementary material for: Patterns of domestication in the Ethiopian oil-seed crop noug (Guizotia abyssinica)
Source: Evol Appl. 2015 Apr 13;8(5):464–75. doi: 10.1111/eva.12256 (PMC4430770; doi:10.1111/eva.12256)
Supplement: Supplementary file 1 — Figure S1. Comparison of STRUCTURE analysis of microsatellite data for the dataset that included all 29 noug accessions and 4 populations of the wild relative, G. scabra ssp schimperii. Figure S2. Comparison of model fit for different numbers of clusters in STRUCTURE analysis for 29 noug populations. Figure S3. Probability intervals for STRUCTURE analysis. Table S1. Summary statistics for noug and its putative wild progenitor, G. scabra ssp schimperii. Table S2. Summary of locus-specific analyses of diversity and divergence. [file eva0008-0464-sd1.docx]

Supp. Table 1. Summary statistics for noug and its putative wild progenitor, *G. scabra* ssp *schimperii*. The following statistics are averaged across populations within each taxon: N, number of individuals genotyped; Na, the number of alleles; I, Shannon-Weaver diversity index; Ho, observed heterozygosity; He, expected heterozygosity; UHe, unbiased expected heterozygosity; F, inbreeding coefficient. PhiST, a measure of divergence among populations, was estimated via analysis of molecular variance (AMOVA) and tested with 1000 random permutations. ‘**’ < 0.01; ‘*’ < 0.05.

|  | Noug |  |  |  |  |  |  | *G. scabra* ssp *schimperii* | | |  |  |  |  |
| --- | --- | --- | --- | --- | --- | --- | --- | --- | --- | --- | --- | --- | --- | --- |
|  | **Na** | **Ne** | **Ho** | **He** | **UHe** | **F** | **PhiST (AMOVA)** | **Na** | **Ne** | **Ho** | **He** | **UHe** | **F** | **PhiST (AMOVA)** |
| **GA003** | 2.517 | 1.331 | 0.183 | 0.235 | 0.243 | 0.226 | 0.01 | 2.667 | 1.759 | 0.236 | 0.426 | 0.439 | 0.446 | 0.046 |
| **GA035** | 3.517 | 1.414 | 0.291 | 0.280 | 0.290 | -0.031 | 0.168** | 3.667 | 2.321 | 0.579 | 0.569 | 0.584 | -0.025 | 0.052* |
| **GA081** | 3.276 | 2.200 | 0.494 | 0.540 | 0.559 | 0.088 | 0.097** | 1.333 | 1.017 | 0.017 | 0.016 | 0.017 | -0.026 | 0 |
| **GA082** | 2.172 | 1.142 | 0.075 | 0.116 | 0.120 | 0.269 | 0.078** | 3.000 | 2.112 | 0.267 | 0.483 | 0.495 | 0.450 | 0.13* |
| **GA108** | 4.172 | 2.420 | 0.389 | 0.574 | 0.591 | 0.318 | 0.079** | 3.333 | 1.563 | 0.239 | 0.357 | 0.366 | 0.331 | -0.017 |
| **GA117** | 2.103 | 1.471 | 0.210 | 0.306 | 0.315 | 0.321 | 0.006 | 3.667 | 2.344 | 0.517 | 0.565 | 0.579 | 0.100 | 0.079* |
| **GA012** | 2.586 | 1.315 | 0.225 | 0.230 | 0.237 | 0.014 | 0.025* | 2.333 | 1.204 | 0.150 | 0.166 | 0.171 | 0.060 | -0.007 |
| **GA138** | 5.414 | 2.903 | 0.481 | 0.649 | 0.668 | 0.257 | -0.005 | 5.667 | 4.019 | 0.633 | 0.726 | 0.744 | 0.127 | 0.123** |
| **GA139** | 5.414 | 2.912 | 0.609 | 0.640 | 0.657 | 0.055 | 0.018* | 4.000 | 2.134 | 0.467 | 0.528 | 0.541 | 0.115 | 0.128* |
| **GA150** | 5.793 | 3.838 | 0.594 | 0.733 | 0.760 | 0.191 | 0.083** | 3.333 | 1.541 | 0.183 | 0.349 | 0.358 | 0.429 | -0.042 |
| **GA029** | 3.828 | 2.366 | 0.486 | 0.560 | 0.578 | 0.118 | 0.073** | 3.333 | 2.519 | 0.683 | 0.596 | 0.612 | -0.145 | 0.028 |
| **GA107** | 3.552 | 1.608 | 0.196 | 0.339 | 0.349 | 0.421 | 0.036** | 3.000 | 2.824 | 0.350 | 0.645 | 0.661 | 0.452 | 0.013 |
| **GA156** | 4.586 | 2.393 | 0.551 | 0.576 | 0.593 | 0.043 | 0.058** | 5.667 | 2.767 | 0.344 | 0.632 | 0.651 | 0.439 | -0.007 |
| **GA162** | 4.138 | 1.783 | 0.391 | 0.421 | 0.433 | 0.058 | 0.054** | 3.000 | 1.344 | 0.117 | 0.248 | 0.255 | 0.550 | -0.022 |
| **GA182** | 7.828 | 3.168 | 0.613 | 0.652 | 0.671 | 0.057 | 0.051** | 3.000 | 2.310 | 0.103 | 0.441 | 0.478 | 0.766 | 0.026 |
| **GA210** | 4.828 | 2.364 | 0.580 | 0.563 | 0.581 | -0.033 | 0.085** | 4.667 | 2.331 | 0.567 | 0.563 | 0.578 | -0.007 | 0.047 |
| ***Total*** | 4.108 | 2.164 | 0.398 | 0.463 | 0.478 | 0.147 | 0.058** | 3.479 | 2.132 | 0.341 | 0.457 | 0.470 | 0.255 | 0.046** |

Supp. Table 2. Summary of locus-specific analyses of diversity and divergence. Shannon’s indices of diversity (allelic information) within noug (sHa_Noug) and *G. scabra* ssp *schimperii* (sHa_CWR), total diversity (sHu) and mutual information or shared diversity (sHua) are described by Sherwin *et al.* (2006). A test of divergence between the taxa using these statistics was highly significant for each locus (G). Analysis of molecular variance (AMOVA) was used to partition genetic variance into components among taxa (PhiRT), among populations within taxa (PhiPR) and among populations relative to the total variance (PhiPT) and tested using 1000 random permutations of the data at each level. *Ia*, the information for ancestry estimation, was calculated using *infocalc* (Rosenberg et al 2003).

|  | **sHa_Noug** | **sHa_CWR** | **sHu** | **sHua** | **G** | **DF** |  | **PhiRT** | **PhiPR** | **PhiPT** | I_a |
| --- | --- | --- | --- | --- | --- | --- | --- | --- | --- | --- | --- |
| **GA003** | 0.497 | 0.711 | 0.534 | 0.009 | 19.717 | 3.000 | *** | 0.027* | 0.014 | 0.041** | 0.006 |
| **GA035** | 0.648 | 1.019 | 0.949 | 0.252 | 576.649 | 8.000 | *** | 0.547** | 0.155** | 0.617** | 0.135 |
| **GA081** | 0.972 | 0.038 | 1.141 | 0.302 | 672.461 | 7.000 | *** | 0.598** | 0.1** | 0.638** | 0.124 |
| **GA082** | 0.292 | 0.829 | 0.432 | 0.072 | 174.043 | 6.000 | *** | 0.223** | 0.092** | 0.295** | 0.028 |
| **GA108** | 1.148 | 0.686 | 1.266 | 0.179 | 425.470 | 8.000 | *** | 0.432** | 0.075** | 0.475** | 0.097 |
| **GA117** | 0.536 | 1.144 | 0.844 | 0.229 | 563.802 | 6.000 | *** | 0.547** | 0.019* | 0.556** | 0.092 |
| **GA012** | 0.463 | 0.283 | 0.456 | 0.016 | 39.336 | 5.000 | *** | 0.054** | 0.027** | 0.079** | 0.008 |
| **GA138** | 1.390 | 1.622 | 1.499 | 0.079 | 193.965 | 14.000 | *** | 0.067** | 0.006 | 0.073** | 0.039 |
| **GA139** | 1.386 | 1.165 | 1.502 | 0.144 | 366.144 | 7.000 | *** | 0.395** | 0.035** | 0.417** | 0.098 |
| **GA150** | 1.618 | 0.732 | 1.550 | 0.061 | 134.213 | 7.000 | *** | 0.182** | 0.08** | 0.247** | 0.044 |
| **GA029** | 1.106 | 1.073 | 1.130 | 0.029 | 70.702 | 8.000 | *** | 0.152** | 0.074** | 0.214** | 0.022 |
| **GA107** | 0.763 | 1.153 | 0.902 | 0.090 | 227.028 | 6.000 | *** | 0.314** | 0.034** | 0.337** | 0.038 |
| **GA156** | 1.167 | 1.356 | 1.208 | 0.018 | 44.595 | 10.000 | *** | 0.023* | 0.046** | 0.069** | 0.012 |
| **GA162** | 0.919 | 0.768 | 1.007 | 0.107 | 273.659 | 7.000 | *** | 0.575** | 0.07** | 0.605** | 0.062 |
| **GA182** | 1.673 | 1.871 | 1.698 | 0.015 | 33.896 | 14.000 | ** | 0.366** | 0.056** | 0.401** | 0.041 |
| **GA210** | 1.254 | 1.336 | 1.395 | 0.131 | 320.891 | 12.000 | *** | 0.359** | 0.092** | 0.418** | 0.076 |
| ***Total*** | 0.989 | 0.987 | 1.095 | 0.108 |  |  |  | 0.358** | 0.061** | 0.397** |  |

Supp. Fig. 1: Comparison of STRUCTURE analysis of microsatellite data for the dataset that included all 29 noug accessions and 4 populations of the wild relative, *G. scabra* ssp *schimperii*. a) Mean log-probability of the data given K clusters, using 20 replicate runs at each value of K between 1 and 20. b) Estimates of Delta K, which can be used as an indicator of the optimal value of K given the data, as described by Evanno et al. (2005).

a


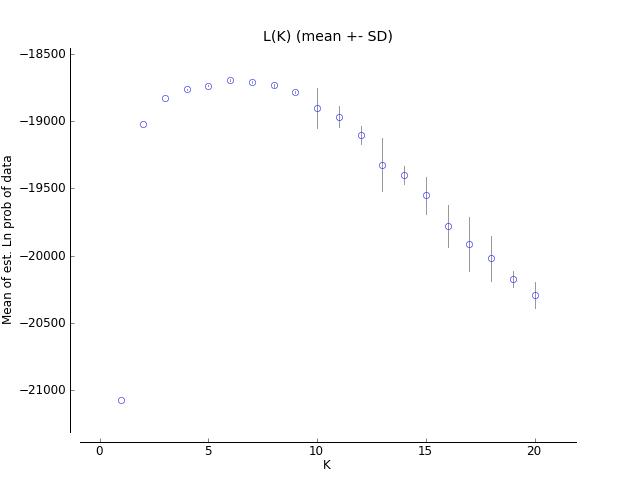


b
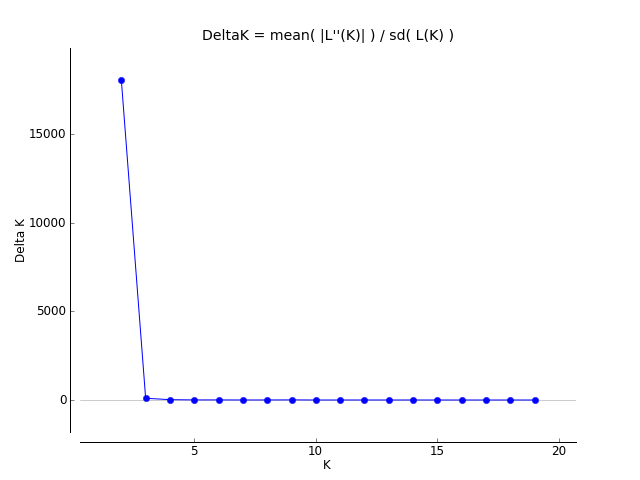


Supp. Fig. 2: Comparison of model fit for different numbers of clusters in STRUCTURE analysis for 29 noug populations. Mean log-probability of the data given K clusters, using 20 replicate runs at each value of K between 1 and 20.
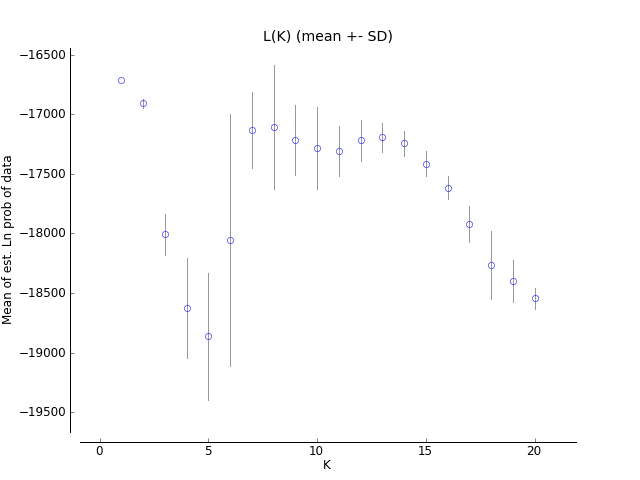


Supp. Fig 3: Probability intervals for STRUCTURE analysis.
